# Relapsing Fever

**Published:** 1852-10

**Authors:** Austin Flint


*Relapsing Fever.* By Professor Austin Flint.—On reviewing the results developed by the foregoing analysis, and comparing them with the distinctive traits of *Relapsing fever*, as presented in the account preliminary to the analysis, the correspondence is certainly striking. The access was abrupt in one-half the cases in which this point was ascertained, and unusually short in duration in the remainder. In 5-14 of the cases, there were no evidences of delirium; and in all the cases, this, together with other head symptoms, was slight.

In 10-15 of the cases there was entire absence of diarrhea, and in one case only was this symptom in any degree prominent or persistent. Vomiting was not a prominent feature being present at the access or commencement in but 8-13, and afterward in but three instances. Iliac tenderness was very slight in every case, and in six cases tenderness over the epigastrium, which is extremely rare in *Typhoid*

and *Typhus* fever, was marked. Meteorism was considerable in but a single instance, and absent in several cases. The abdominal symptoms, thus, were absent or slight in a remarkable degree, unless the cases were of the *Typhus* type, and it is to be borne in mind that none of the cases were considered to be of that type. The chest symptoms, and the circulation offer nothing worthy of particular note, except that the frequency of the pulse was considerably less than is stated to belong to the history of Relapsing fever. Sweating and moisture occurred in a very large proportion of the cases, in this respect presenting a contrast with the average occurrence of these symptoms in *Typus* and *Typhoid* fever; but differing from other observations with respect to *Relapsing* fever in the fact that these symptoms did not occur at the time of the temporary or permanent convalescence. Mild jaundice, a very rare event in *Typhus* or *Typhoid* fever, was present in *two* of the fifteen cases. But the most striking of all the points of correspondence relate, first, to eruptions, and, second, to the relapses. In none of the cases was there an eruption, a fact which would certainly be very remarkable with respect to the same number of cases of the *Typhus* or *Typhoid* type occurring successively. The circumstances pertaining to the relapses, viz., the duration of the first febrile career, of the interval, and of the second febrile attack, accord with the previous description of these events as they are stated to occur in *Relapsing fever.* Finally, the absence of a fatal tendency is exemplified by the fact that all the cases ended in recovery.

In view of these facts the conclusion seems unavoidable that the cases of fever characterized by relapses, among those which came under my observation in 1850–'51, presented the distinctive traits attributed to *Relapsing fever* sufficiently marked to entitle them to be ranked in the class of cases which have been described by different observers as a peculiar form of Continued Fever. This conclusion does not necessarily involve the position that the traits distinguishing the cases authorize their separation as cases of an affection entirely distinct from *Typhus* or *Typhoid* fever. It of course follows either that this position is correct, or that the *Typhoid* or *Typhus* forms of Continued Fever occasionally exhibit, as peculiar modifications, the symptoms which are considered the diagnostic features of *Relapsing fever* by those who regard the latter as a separate form of the disease. I am not prepared to discuss the relative merits of these inferences.—*Buffalo Med. Journal.*

